# Changes in Biomarkers of Exposure on Switching From a Conventional Cigarette to the glo Tobacco Heating Product: A Randomized, Controlled Ambulatory Study

**DOI:** 10.1093/ntr/ntaa135

**Published:** 2020-08-10

**Authors:** Nathan Gale, Michael McEwan, Oscar M Camacho, George Hardie, James Murphy, Christopher J Proctor

**Affiliations:** British American Tobacco (Investments) Limited, Research and Development, Southampton, UK

## Abstract

**Introduction:**

Tobacco heating products (THPs) generate lower machine yields of toxicants compared to those found in conventional cigarette smoke. During use, these products are likely to expose users to lower levels of particulate matter and harmful and potentially harmful compounds compared with smoking cigarettes.

**Aims and Methods:**

This randomized, controlled study is investigating whether biomarkers of exposure (BoE) to smoke toxicants are reduced when smokers switch from smoking cigarettes to using the glo THP in a naturalistic, ambulatory setting. Control groups include smokers who are abstaining from cigarette smoking and never-smokers. At a baseline study visit, 24-hour urine samples and spot blood samples were taken for BoE analysis, and exhaled carbon monoxide was also measured. *N*-(2-cyanoethyl) valine (CEVal) was used as a marker of compliance in subjects asked to refrain from combustible cigarette smoking. Subjects are being followed up at periodic intervals for 360 days; this article presents data following a planned interim analysis at day 90.

**Results:**

In continuing smokers, BoE remained stable between baseline (day 1) and day 90. In both per-protocol and CEVal-compliant analysis populations, reductions in BoE were observed in subjects switching to using glo or undergoing smoking cessation. These reductions were statistically significant for a number of BoE when switching to glo was compared with continued smoking. Furthermore, in both populations, reductions observed in subjects switching to using glo were comparable to those seen with smoking cessation and were also to levels similar to those seen in never-smokers.

**Conclusion:**

glo is a reduced-exposure tobacco product.

**Implications:**

This clinical study builds on a previous 5-day confinement study and demonstrates that when smokers switched from smoking combustible cigarettes to using the glo THP in a naturalistic, ambulatory setting, their exposure to tobacco smoke toxicants was significantly decreased. For most BoE examined, this was to the same extent as that seen when a control group of smokers ceased cigarette smoking, or even to levels seen in never-smoker controls. This indicates that glo is a reduced-exposure product with the potential to be a reduced-risk tobacco product, when used by smokers whose cigarette consumption is displaced completely.

**Clinical trial registration:**

ISRCTN81075760.

## Introduction

Cigarette smoking is a leading cause of numerous human disorders including lung cancer, chronic obstructive pulmonary disease, and cardiovascular disease.^1^ Risks of smoking correlate with years since smoking initiation and daily cigarette consumption and are principally due to inhalational exposure to a number of smoke toxicants transferred into cigarette smoke during the combustion of tobacco.^1–7^ Quitting smoking reduces the disease risk,^1^ and as such the public health priority of reducing the health burden of cigarette smoking has led to the development of a variety of initiatives to reduce smoke toxicant exposure by encouraging smoking abstinence.^8^ More recently, however, the question has arisen of whether these public health goals can be met through the development of new nicotine and tobacco products to support combustible cigarette displacement^9^ and reduce/eliminate toxicant exposure.

Of the more than 6500 identified chemical constituents of combustible cigarette smoke,^10^ many have been identified as potential contributors to the harmful effects of smoking.^11–13^ The US Institute of Medicine (IoM) in 2001 proposed the development of potential reduced-exposure products (PREPs) that yield lower emissions of some toxicants compared with conventional, combustible cigarettes and that could be expected to result in reduced toxicant exposure in smokers who completely switch to using such products.^4,5^ The IoM concluded that “For many diseases attributable to tobacco use, reducing risk of disease by reducing exposure to tobacco toxicants is feasible,” thus setting the foundations for a toxicant exposure reduction approach to tobacco harm reduction. Although combustible cigarettes with reduced emissions have been developed,^14,15^ changes in health indicators were not seen following long-term switching from conventional cigarette smoking to using these products.^16^ However, since then, novel tobacco heating products (THPs) that heat rather than burn tobacco have been developed. These deliver nicotine in an inhalable aerosol, but with lower or immeasurable aerosol levels of the toxic constituents associated with combusting tobacco.^17^ As such, THPs exhibit lower machine yields of toxicants compared to those found in conventional cigarette smoke.^18^ Following a review of the literature Public Health England recently concluded that “Compared with cigarette smoke, heated tobacco products are likely to expose users and bystanders to lower levels of particulate matter and harmful and potentially harmful compounds”.^19^ One such product is the glo THP, developed by British American Tobacco. The glo THP electronically heats cylindrical tobacco sticks (“Neostiks”) to a maximum temperature of 240 ± 5°C.^20^ In a clinical study with glo, biomarker measurements showed that exposure to cigarette smoke toxicants in smokers who switched to using glo for 5 days in a confinement setting was reduced to levels seen in subjects who refrained completely from using any tobacco products.^21^

The aim of this current study was to extend the findings of the previous confinement study^21^ and determine whether lowering of toxicant exposure when switching from smoking to using glo was maintained over a longer period of time and in a more naturalistic, ambulatory setting. This article describes findings from a planned interim analysis on a subset of study subjects at day 90 of a 12-month study^22^ in which cigarette smokers either remained smoking, switched to using glo, or abstained completely from tobacco/nicotine product use.

## Methods

A full description of the study protocol has been published previously.^22^ Brief study details are described here.

### Study Design

This is an ongoing, randomized, controlled, parallel-group, open-label, ambulatory clinical study being carried out at four sites in the United Kingdom (Leeds, Belfast, London, and Merthyr Tydfil). The study was registered on the ISRCTN registry (ISRCTN81075760). A favorable opinion was given by a Research Ethics Committee (NHS Health Research Authority, Wales Research Ethics Committee 2; reference number: 17/WA/0212). The study is being conducted in compliance with the ethical principles of the Declaration of Helsinki, Good Clinical Practice (International Council for Harmonisation (ICH) E6 Consolidated Guidance, April 1996), and UK laws, including those relating to the protection of subjects’ personal data. Written informed consent was obtained from all individual subjects prior to their participation in the study and before undergoing any study procedures, including screening assessments.

### Subjects

During a screening visit, potential subjects were assessed for their eligibility based on inclusion/exclusion criteria which have been described in full previously.^22^ In brief, healthy male or female subjects were enrolled in this study, who were aged 23–55 inclusive and in good general health with no clinically relevant abnormal findings on physical examination, vital signs assessment, ECG, clinical laboratory evaluations or lung function tests, or in their medical history. For Groups A, B, and D (remain smoking, switch to glo, or quit any tobacco product use, respectively), current smokers were recruited who self-reported daily smoking of 10–30 non-menthol factory-manufactured or roll-your-own cigarettes and had at least 5 years’ consecutive smoking history. Current smoking was verified using urinary cotinine (>200 ng/mL) and exhaled breath carbon monoxide (eCO; ≥7 ppm) tests. Subjects assigned to Group D self-reported intending to quit smoking, to maximize the potential for compliance in this group. For Group E (never-smokers), subjects self-reported having smoked less than 100 cigarettes in their lifetime and none in the 30 days prior to screening. Nonsmoking status was verified by negative urine cotinine and eCO tests.

Main exclusion criteria were subjects who did not agree, or whose partners of childbearing potential did not agree, to use effective methods of contraception for the duration of the study; female subjects who were pregnant or breast feeding; subjects who had donated at least 400 mL of blood within 12 weeks (males) or 16 weeks (females) prior to study start; subjects who had an acute illness (eg, upper respiratory tract infection) requiring treatment within 4 weeks prior to study start; subjects who regularly used any nicotine or tobacco products other than commercially manufactured filter cigarettes and/or roll-your-own cigarettes within 14 days of screening; subjects who were self-reported non-inhalers (smokers who draw smoke from the cigarette into the mouth and throat but do not inhale); subjects who had received any medications or substances (other than tobacco) which interfere with the cyclooxygenase pathway or are known to be strong inducers or inhibitors of cytochrome P-450 enzymes, within 14 days or 5 half-lives of the drug prior to study start. Subjects were excluded from Groups A and B if they were planning to quit smoking in the next 12 months. All subjects were informed that they were free to quit smoking and withdraw from the study at any time. Any subject who decided to quit smoking was directed to appropriate stop smoking services.

### Investigational Products

Characteristics of the glo device have been published previously,^20^ while aerosol emissions for the Neostiks used in this study can be found in Supplementary Table 1. All study glo devices/Neostiks (Group B) were provided to subjects free of charge. The supply of Neostiks was limited to a maximum of 200% of the subjects’ self-reported daily cigarette consumption at screening. This restriction was made to prevent excessive increases in product consumption which has been reported by ourselves and others in subjects provided with free tobacco products in clinical studies,^16,23^ while allowing for some flexibility in changing product use behavior following switching since the nicotine yield of each Neostik would likely be lower than that of the subject’s usual brand cigarette. Subjects in Group A were required to purchase their own usual brand combustible cigarettes throughout the study. Subjects in Group D had a cessation strategy devised with the Investigator to support cessation, which included nicotine replacement therapy (NRT) and/or varenicline provision if requested, alongside cessation counseling.

A single study product/intervention was allocated to each group; these were subjects’ usual brand of non-menthol factory-made or roll-your-own combustible cigarette (Group A); glo with non-menthol Neostiks (Group B); and complete abstention from nicotine/tobacco product use, other than NRT if provided (Group D). Group E were never-smokers at baseline and throughout the study.

### Study Procedures

A study design schematic has been published previously.^22^ At screening, subjects underwent testing to ensure that they met all inclusion and no exclusion criteria. Subjects also completed a tobacco use history questionnaire and the Fagerström Test for Cigarette Dependence (FTCD).^24^ At Visit 1 (baseline), subjects underwent safety assessments prior to randomization. At this visit, 24-hour urine samples and spot blood samples were taken for BoE analysis, and eCO measurements were also made. After this visit and according to the randomization code, subjects either remained smoking their own brand of combustible cigarettes (Group A), switched to using glo (Group B), or refrained from using any nicotine/tobacco products (Group D). This switching period was scheduled to last for 12 months, and this article reports findings over the initial 90 days of the study. Subjects in Group E (never-smokers) were asked to refrain from initiating the use of any nicotine/tobacco products for the duration of the study.

Subjects in Groups A, B, and D returned to the clinic on days 30, 60, and 90 (for Visits 2, 3, and 4 respectively), at which the same BoE measurements were made as Visit 1. Subjects in Group E only returned to the clinic for a single visit on day 90.

### Compliance

Following screening but before randomization, subjects in Groups A and B were offered the opportunity to try up to two Neostiks, to allow subjects to experience the product they could be randomized to use. Subjects could decide whether to continue to participate in the study following this trial. Subjects were instructed of the importance of complying with exclusive use of their randomized product (Groups A and B) or of not smoking cigarettes/using nicotine products (Groups D and E). Subjects were asked to report any noncompliance using electronic and paper diaries and were informed that compliance assessments would be conducted; this was achieved by measuring levels of a hemoglobin adduct of acrylonitrile (*N*-(2-cyanoethyl) valine [CEVal]) in all study participants as a marker of combusted tobacco exposure. Acrylonitrile is below the detection limit in glo emissions and has no common environmental source, but is found in cigarette smoke. Thresholds for CEVal used to deduce compliance were calculated based on a previous study where this biomarker was reported for a modified combustible prototype cigarette.^16,25^

### Statistical Methods

A full statistical analysis plan including sample size determination methods has been published previously.^25^ In summary, BoE levels were computed at each timepoint, and the levels at day 90 were compared between the glo group (B) and the continued smoking group (A) using specific contrast tests from statistical models adjusted for baseline measurements. Alpha level across timepoints was adjusted using the O’Brien-Fleming approach,^26^ with 0.0006 overall alpha available at day 90. Multiplicity adjustment for family-wise error was performed using Holm’s method.^27^ A planned interim analysis was performed on those subjects who were enrolled on or before the day the 42nd subject was enrolled into the continue to smoke study group and who were still participating at day 90. This cutoff point was chosen aiming to provide a minimum of 30 subjects in Group A still enrolled in the study at day 90 to give sufficient power to detect a statistical difference between Groups A and B for total 4-(methylnitrosamino)-1-(3-pyridyl)-1-butanol (NNAL), the primary BoE endpoint.

Missing values were not imputed and values below the analytical limit of detection (LOD) or lower limit of quantification (LLOQ) were replaced with half the value of the LOD or LLOQ, respectively. Data analysis was performed using SAS version 9.4.

### Analysis Populations

The per-protocol (PP) population includes all subjects who had a valid assessment of a biomarker variable and completed the study (to day 90) according to the protocol. This population excludes subjects in Groups B and D who had major protocol deviations or a significant level of self-reported smoking. The CEVal-compliant population excludes subjects in Groups B and D who were considered noncompliant with smoking restrictions, based on CEVal levels above predetermined thresholds.^25^

### Biomarkers of Exposure

Biomarkers of exposure (BoE) to selected cigarette smoke constituents in 24-hour urine collections were measured at baseline and days 30, 60, and 90. The study is examining the following urinary BoE: total nicotine equivalents (TNeq; nicotine, cotinine, 3-hydroxycotinine and their glucuronide conjugates); total NNAL; total *N*-nitrosonornicotine (NNN); 3-hydroxypropylmercapturic acid (3-HPMA); 3-hydroxy-1-methylpropylmercapturic acid (HMPMA); *S*-phenylmercapturic acid (S-PMA); monohydroxybutenyl-mercapturic acid (MHBMA); 2-cyanoethylmercapturic acid (CEMA); 4-aminobiphenyl (4-ABP); *o*-toluidine (*o*-Tol); 2-aminonaphthalene (2-AN); 1-hydroxypyrene (1-OHP); and 2-hydroxyethylmercapturic acid (HEMA). CO was measured in exhaled breath, and the compliance biomarker CEVal was measured in whole blood. For details of the smoke constituent associated with each BoE, and details of the LOD, LLOQ, and ULOQ for each BoE measured, see Supplementary Table 2.

Laboratory analyses of urine and blood biomarkers were carried out at ABF GmbH (Planegg, Germany). Details of the bioanalytical methods have been published previously.^21^

## Results

### Participant Demographics

Basic participant demographic details are presented in Table 1; overall gender split was 55:45 males to females with some minor differences between groups. Baseline cigarette consumption was broadly similar between Groups A, C, and D, as was the total FTCD score. Subjects were predominantly white (between 87.5% and 92.0%) in each study group, and there were no notable differences in age, weight, or body mass index between study groups.

**Table 1.** Demographic Data for Study Participants

		Group				
		A (continue to smoke)	B (switch to glo)	D (cessation)	E (never-smoker)	Overall
*N* (enrolled)		42	105	190	40	377
*N* (interim PP population)	*N* (% of enrolled)	32 (76.2%)	75 (71.4%)	136 (71.6%)	37 (92.5%)	280 (74.3%)
Age (years)	Mean (*SD*)	38 ± 9.3	39 ± 8.8	38 ± 9.0	40 ± 9.9	39 ± 9.1
Sex	Male:Female	1.46:1	1.03:1	1.52:1	0.68:1	1.22:1
Weight (males; kg)	Mean (*SD*)	79.7 ± 10.32	79.6 ± 11.60	82.1 ± 11.33	80.0 ± 11.64	81.0 ± 11.26
Weight (females; kg)	Mean (*SD*)	63.0 ± 10.36	68.5 ± 9.43	65.2 ± 9.30	66.1 ± 8.51	66.1 ± 9.38
BMI (kg/m^2^)	Mean (*SD*)	25.2 ± 3.16	25.3 ± 3.30	25.4 ± 2.97	25.4 ± 3.04	25.4 ± 3.08
FTCD total score	Mean (*SD*)	5 ± 1.8	6 ± 1.7	5 ± 1.8	N/A	5 ± 1.8
Cigarettes/day^a^	Mean (*SD*)	18 ± 5.5	18 ± 5.5	18 ± 5.3	N/A	18 ± 5.4
Race	*N* (%)					
Asian		1 (3.1%)	4 (5.3%)	3 (2.2%)	2 (5.4%)	10 (3.6%)
Black/African American		2 (6.3%)	0 (0%)	3 (2.2%)	2 (5.4%)	7 (2.5%)
White		28 (87.5%)	69 (92.0%)	124 (91.2%)	33 (89.2%)	254 (90.7%)
Other		1 (3.1%)	2 (2.7%)	6 (4.4%)	0 (0%)	9 (3.2%)
Ethnicity	*N* (%)					
Hispanic/Latino		0 (0%)	1 (1.3%)	2 (1.5%)	0 (0%)	3 (1.1%)
Not Hispanic/Latino		32 (100%)	74 (98.7%)	134 (98.5%)	37 (100%)	277 (98.9%)

BMI, body mass index; *N*, number of subjects; PP, per protocol; FTCD, Fagerström Test for Cigarette Dependence.

^a^Self-reported cigarette consumption at screening.

### Cigarette and glo Neostik Consumption

In Group A, cigarette consumption at all timepoints up to day 90 (Supplementary Table 3) remained largely similar to those self-reported by subjects at screening (Table 1). In Group B, the numbers of Neostiks used per day were slightly higher at all timepoints (Supplementary Table 3) than usual brand combustible cigarette consumption reported at screening (Table 1) but remained stable between baseline and day 90.

### Biomarkers of Exposure

**Figure 1.**
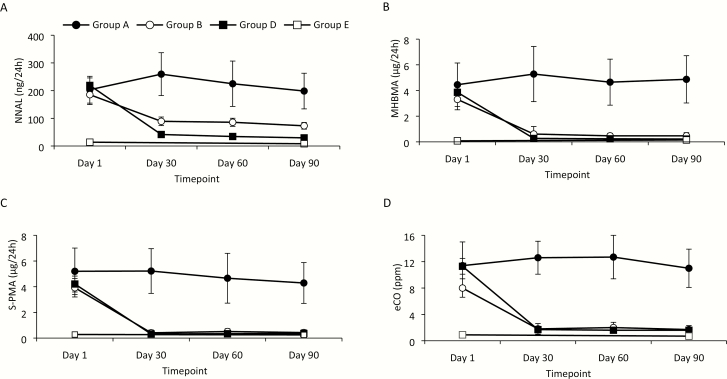
Changes in biomarkers of exposure over time in the CEVal-compliant population. Plots show absolute levels of 24-hour urinary excretion of (A) 4-(methylnitrosamino)-1-(3-pyridyl)-1-butanol (NNAL), (B) monohydroxybutenyl-mercapturic acid (MHBMA), (C) *S*-phenylmercapturic acid (S-PMA), and (D) exhaled breath carbon monoxide (eCO) at baseline (day 1, all groups) and at days 30, 60, and 90 (smokers at baseline [Groups A, B, and D]) or at day 90 (never-smokers [Group E]). Data are presented as means ± CI in subjects in the CEVal-compliant population who continued to smoke combustible cigarettes (Group A; *n* = 32), switched to using glo (Group B; *n* = 60), abstained from cigarette smoking (Group D; *n* = 107), or were never-smokers (Group E; *n* = 35). The legend in panel A is applicable to all other panels.

In Group A (continue to smoke), BoE remained stable between baseline (day 1) and day 90; Figure 1 shows example time-series plots for NNAL, MHBMA, S-PMA, and eCO. For each BoE assessed in Group B (switching to glo) of the CEVal-compliant population, reductions were observed between day 1 and day 30, and these were sustained at the same level between day 30 and day 90. For some biomarkers, these reductions reached levels approximating to those seen in Group D (cessation, CEVal-compliant population) and also close to levels observed in Group E (never-smokers; Figure 1).

**Figure 2.**
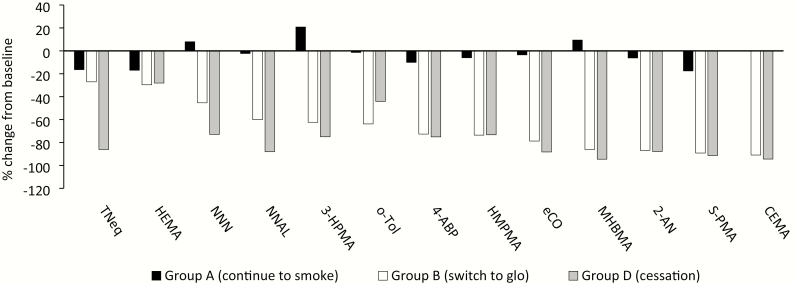
Mean percentage changes in biomarkers of exposure between baseline (day 1) and day 90 in the CEVal-compliant population. Data are mean values expressed as a percentage of the baseline value. All data, except for eCO, were calculated using biomarker levels from 24-hour urine collections at baseline (day 1) and on day 90. eCO change was calculated from data captured at a single timepoint at baseline and on day 90; *n* = 32 (Group A), 60 (Group B), and 107 (Group D). TNeq, total nicotine equivalents (nicotine, cotinine, 3-hydroxycotinine and their glucuronide conjugates); HEMA, 2-hydroxyethylmercapturic acid; NNN, *N*-nitrosonornicotine; NNAL, 4-(methylnitrosamino)-1-(3-pyridyl)-1-butanol; 3-HPMA, 3-hydroxypropylmercapturic acid; *o*-tol, *o*-toluidine; 4-ABP, 4-aminobiphenyl; HMPMA, 3-hydroxy-1-methylpropylmercapturic acid; eCO, exhaled carbon monoxide; MHBMA, monohydroxybutenyl-mercapturic acid; 2-AN, 2-aminonaphthalene; S-PMA, *S*-phenylmercapturic acid; CEMA, 2-cyanoethylmercapturic acid.

Changes in BoE were examined between baseline (day 1) and day 90 in the CEVal-compliant population (Figure 2). Broadly speaking, in Group A BoE remained stable between these timepoints, though some moderate positive and negative changes from baseline were seen for some BoE. In Group B, all BoE were lower at day 90 compared with baseline; average reductions ranged from −27.0% for TNeq to −90.9% for CEMA (Figure 2), with some reaching levels approximating those seen in the cessation group (D) and close to levels observed in never-smokers (Group E). When compared to the differences between baseline and day 90 in the continued smoking group (Table 2), the reductions in the glo group were statistically significant (99.94% CI; *p* < .0001) for NNAL, 3-HPMA, 4-ABP, HMPMA, eCO, MHBMA, 2-AN, S-PMA, and CEMA. Despite the mean reductions from baseline being in line with cessation for HEMA and *o*-Tol, and NNN reducing over half as much as seen for cessation, statistical significance was not reached for these BoE following multiple-comparison adjustment. Change from baseline to day 90 for nicotine exposure, assessed by TNeq, was not significantly different (*p* = .34) between Groups A and B, since in both these groups TNeq was reduced by a similar amount, around 20%, between these study timepoints.

**Table 2.** Between-Group Statistical Analysis of Change From Baseline (Day 1) to Day 90 in Biomarkers of Exposure in the CEVal-Compliant Population

Biomarker (units)	Study group	*N*	LS mean	Comparison	Difference (99.94% CI)	*p*
TNeq (mg/24 h)	Group A	32	−2.83	B – A	−1.36 (−6.36, 3.65)	.33810
	Group B	60	−4.18			
HEMA (µg/24 h)	Group A	32	−2.21	B – A	−1.12 (−10.09, 7.86)	.65940
	Group B	60	−3.33			
Total NNN (ng/24 h)	Group A	32	1	B – A	−4.61 (−25.77, 16.56)	.44070
	Group B	60	−3.61			
Total NNAL (ng/24 h)	Group A	32	−5	B – A	−105 (−193, −17)	<.0001
	Group B	60	−110			
3-HPMA (µg/24 h)	Group A	32	249	B – A	−926 (−1426, −426)	<.0001
	Group B	60	−677			
*o*-tol (ng/24 h)	Group A	32	−2	B – A	−112 (−257, 33)	.00730
	Group B	60	−114			
4-ABP (ng/24 h)	Group A	32	−2	B – A	−9.5 (−15.9, −3.1)	<.0001
	Group B	60	−11.6			
HMPMA (µg/24 h)	Group A	32	−28	B – A	−285 (−440, −130)	<.0001
	Group B	60	−313			
eCO (ppm)	Group A	32	14.98	B – A	−13.38 (−17.94, −8.82)	<.0001
	Group B	60	1.61			
MHBMA (µg/24 h)	Group A	32	0.42	B – A	−3.34 (−5.62, −1.07)	<.0001
	Group B	60	−2.92			
2-AN (ng/24 h)	Group A	32	−1.7	B – A	−17.6 (−26.9, −8.4)	<.0001
	Group B	60	−19.4			
S-PMA (µg/24 h)	Group A	32	−0.91	B – A	−2.64 (−4.79, −0.5)	<.0001
	Group B	60	−3.55			
CEMA (µg/24 h)	Group A	32	0	B – A	−151 (−219, −83)	<.0001
	Group B	60	−151			

TNeq, total nicotine equivalents (nicotine, cotinine, 3-hydroxycotinine and their glucuronide conjugates); HEMA, 2-hydroxyethylmercapturic acid; NNN, *N*-nitrosonornicotine; NNAL, 4-(methylnitrosamino)-1-(3-pyridyl)-1-butanol; 3-HPMA, 3-hydroxypropylmercapturic acid; *o*-tol, *o*-toluidine; 4-ABP, 4-aminobiphenyl; HMPMA, 3-hydroxy-1-methylpropylmercapturic acid; eCO, exhaled carbon monoxide; MHBMA, monohydroxybutenyl-mercapturic acid; 2-AN, 2-aminonaphthalene; S-PMA, *S*-phenylmercapturic acid; CEMA, 2-cyanoethylmercapturic acid; LS mean, least squares mean; CI, confidence interval.

Group A, continue to smoke combustible cigarettes; Group B, switch to glo. All analyses, except for eCO, were performed using biomarker levels from 24-h urine collections at baseline (day 1) and on day 90. Statistical analysis for the change in eCO levels was performed on data captured at baseline and the average of values obtained on days 120 and 150.

In the PP population, percentage reductions from baseline (Supplementary Figure 1) in subjects in Group B (switch to glo) were largely similar to those seen in the CEVal-compliant population (Figure 2). Furthermore, in the PP population, BoE levels in Group B reached levels at day 90 approximating those seen in Group D (cessation) and close to levels observed in never-smokers (Group E). As per the CEVal-compliant population, differences between baseline and day 90 between Groups A and B (Supplementary Table 4) were statistically significant for NNAL, 3-HPMA, 4-ABP, HMPMA, eCO, MHBMA, 2-AN, S-PMA, and CEMA, and not for HEMA, *o*-Tol, and NNN following multiple-comparison adjustments and despite average reductions being similar to or even greater than cessation. TNeq was similar in the PP population in subjects continuing to smoke or switching to glo.

## Discussion

For combustible cigarette smokers, inhalational exposure to smoke toxicants is a contributor to the harmful effects of cigarette smoking, and therefore efforts to reduce this exposure is a potential approach to tobacco harm reduction.^4,5^ In this article, we describe data between baseline and day 90 of an ambulatory switching study in which current smokers were randomly selected to either remain smoking cigarettes, to switch to using the glo THP, or to quit the use of any tobacco/nicotine product. Assessments of exposure were made using urinary and breath BoE. In continuing smokers and as expected, average BoE levels remained constant between baseline and day 90. In those subjects who switched to glo, however, and both in a PP population and in a cohort of subjects whose abstention from smoking cigarettes was biochemically verified using CEVal, significant and sustained reductions were observed for a number of BoE for toxicants with a known link to smoking-related disease.^11^ Importantly, these reductions were similar in magnitude to those seen in the group of subjects who abstained from tobacco product use, and furthermore, the levels at day 90 for a number of BoE approached those seen in a control group of never-smokers.

Similar to this current study, long-term post-switching exposure to carbon monoxide has been reported to be reduced to levels close to those seen in nonsmokers.^28^ Furthermore, this study builds on a previous 5-day confinement study^21^ and provides evidence that switching to the glo THP can reduce smokers’ exposure to many harmful cigarette smoke toxicants. By reporting data in both noncompliant and compliant populations of subjects, we are able to demonstrate estimations of reductions in exposure both in a real-world scenario and when the glo THP is used to fully displace cigarette smoking. It is important to note that the BoE reductions were observed despite both tobacco product consumption and nicotine exposure being sustained and comparable in those subjects who switched to using glo compared to those who remained smoking combustible cigarettes.

While our findings do not confirm any reduction in risk when switching to glo compared to remaining smoking combustible cigarettes, they are in accordance with Public Health England’s suggestion that heated tobacco products may be less harmful than combustible tobacco cigarettes,^19^ and with the work of other groups that have demonstrated reductions in toxicant exposure in smokers switching to using THPs in long-term ambulatory studies.^28–30^ Importantly, long-term reductions in combustible cigarette toxicant exposure in people who switch from smoking cigarettes to using heated tobacco products have been reported to be associated with favorable changes in biomarkers of biological effect, which included indicators of inflammation, oxidative stress, cardiovascular and pulmonary health, and lung cancer risk.^30^ While not reported here, this current study is also examining a wider range of health effect indicators associated with smoking-related disease development than previous studies and over a longer (12-month) period of switching.^22,25^ Future analyses will therefore reveal whether the sustained reductions in toxicant exposure seen when switching to glo and reported here also lead to beneficial changes in biological effect biomarkers.

Although directionally consistent, not all BoE reductions in the glo group were statistically significant when compared to the change in the continued smoking group, despite robust reductions in these BoE over the 90-day assessment period and in the absence of corresponding emissions from glo (Supplementary Table 1 and the study of Forster et al.^18^). For example, HEMA was reduced in subjects switching to using glo in line with reductions also seen in the smoking cessation group. However, the reduction in HEMA BoE levels in the continued smoking group (by around 20% between baseline and day 90) is likely the cause of the lack of statistical significance. Regarding *o*-toluidine, average reductions in subjects switching to glo were greater than those seen in the smoking cessation group. However, higher variability, especially in Group A, led to lack of statistical significance for this group comparison. The reasons for this are unclear, though environmental exposure from food and chemicals (eg, hair dyes) may provide an auxiliary source of exposure to aromatic amines such as *o*-toluidine.^31,32^

While the BoE for the two tobacco-specific nitrosamine BoE examined were reduced in those switching to glo between baseline and day 90, when compared to continued smoking this was statistically significant for the NNK BoE, NNAL, but not for NNN. It is noteworthy that in machine-derived emissions from glo compared to those found in cigarette smoke, reductions in NNN are lower than those for NNK,^18^ and this may provide a potential explanation for the nonsignificant reduction in the NNN BoE found in this study. Furthermore, a recently published study examining changes in BoE in smokers who switched to using e-cigarettes^33^ raised the notion that, at least in some subjects and in the absence of inhaled NNN, urinary NNN excretion could be due to endogenous nitrosation of nicotine and/or nornicotine in acidic environments (eg, stomach), or in the presence of bacteria that catalyze nitrosation at neutral pH (eg, oral cavity).^34^ Sporadic endogenous formation of NNN and its appearance in urine have also been reported for users of oral and dermal NRT products.^35,36^ Further studies are required to identify the source of NNN in the urine, as well as to determine whether its presence is artefactual in nature, whether it is produced endogenously in people who switch to using glo, and whether its presence has any deleterious health implications.

While this ambulatory study design builds on our previous confinement study^21^ in providing real-world evidence of exposure reductions in smokers switching to glo, there are still some limitations. Primarily, by limiting our analyses to PP and CEVal-compliant populations, the findings do not indicate likely changes in population-level exposure and particularly in those who dual use cigarettes and glo. However, in this study, a large proportion of subjects had completely switched to using glo and the findings may therefore be indicative of changes in a broader population. Furthermore, future planned analyses on the PP population and the total population (ie, those subjects who underwent randomization and regardless of protocol noncompliance) will give an indication of real-world exposure changes in subjects who dual use glo and combustible cigarettes. Secondly, while reductions in BoE were observed for a number of smoke toxicants linked with smoking related diseases, the study design precludes drawing conclusions on reductions in exposure to smoke toxicants other than those for which BoE were examined in this study. Importantly, while exposure reduction is demonstrated, this cannot be extrapolated to any reduction in disease risk. However, future analyses of biomarker of biological effect data being collected in this study may provide indications of any such reductions in risk in smokers who switch to using glo. Finally, this study did not collect data concerning changes in the usual brand of cigarettes smoked, occupation, or other environmental changes that could explain the modest changes or variability in some BoE observed in continued smokers, which may have led to the lack of significance in intergroup comparisons.

In conclusion, this study demonstrates in a naturalistic, real-world setting that glo is a reduced-exposure product with the potential to be a reduced-risk tobacco product. While no assessment of the potential impact of our findings on health risks can be made at this stage of the study, our findings clearly demonstrate the potential for the deleterious health impacts of cigarette smoking to be reduced in smokers who completely switch to using glo. Future analyses on study completion will indicate whether reduced exposure is maintained over a period of 1 year. Furthermore, analysis of study data arising from the measurement of health effect indicators (biomarkers of potential harm) will facilitate an assessment of whether the observed reductions in exposure are accompanied by potential reductions in the health risks associated with cigarette smoking.

## Supplementary Material

A Contributorship Form detailing each author’s specific involvement with this content, as well as any supplementary data, is available online at https://academic.oup.com/ntr

ntaa135_suppl_Supplementary_Table_1

ntaa135_suppl_Supplementary_Table_2

ntaa135_suppl_Supplementary_Table_3

ntaa135_suppl_Supplementary_Table_4

ntaa135_suppl_Supplementary_Figure_1

ntaa135_suppl_Supplementary_Taxonomy_Form
