# Supplementary material for: Changes in Biomarkers of Exposure on Switching From a Conventional Cigarette to the glo Tobacco Heating Product: A Randomized, Controlled Ambulatory Study
Source: Nicotine Tob Res. 2020 Aug 10;23(3):584–91. doi: 10.1093/ntr/ntaa135 (PMC7885769; doi:10.1093/ntr/ntaa135)
Supplement: ntaa135_suppl_Supplementary_Table_1 [file ntaa135_suppl_supplementary_table_1.docx]

Supplementary Table 1. Aerosol Emissions Data for glo Compared to 3R4F Reference Cigarette Smoke. Values are machine yields of numerous cigarette smoke toxicants, when 3R4F University of Kentucky reference cigarettes were smoked to the designated butt-mark using the standard Health Canada intense smoking regime (55 ml puff volume, 2 s puff duration, 30 s puff interval, bell-shaped puff profile and 100% ventilation blocking) and glo and Neostiks were used for 9 puffs under the same regime without vent blocking. Two distinct batches of glo Neostiks were manufactured for use in the study; data for each batch are presented. 3R4F Batch 1 TobReg9 data are based on three analytical replicates whereas all other TobReg9 and aromatic amines data are based on five analytical replicates. For certain compounds, the value reported was below the limit of quantification (LOQ) or below the limit of detection (LOD). When this occured, a derived value was used for the percentage reduction calculation. When the value was <LOD, half the LOD value was used. When the value was <LOQ, the midpoint between the LOD and LOQ was used. BDL, below detection limit, with LOD shown in parentheses; NQ, not quantifiable, with LOQ shown in parentheses; NC, not calculated; SD, standard deviation; TobReg9, World Health Organization (WHO) Study Group on Tobacco Product Regulation cigarette smoke toxicants recommended for mandated lowering; CO, carbon monoxide; NNN, N‑nitrosonornicotine; NNK, 4-(Methylnitrosamino)-1-(3-pyridyl)-1-butanone.

|  |  | **3R4F** | | **Neostik Batch 1** | | | **3R4F** | | **Neostik Batch 2** | | |
| --- | --- | --- | --- | --- | --- | --- | --- | --- | --- | --- | --- |
| **Constituent** | **Unit** | **Average** | **SD** | **Average** | **SD** | **% reduction** | **Average** | **SD** | **Average** | **SD** | **% reduction** |
| **TobReg9** |  |  |  |  |  |  |  |  |  |  |  |
| CO | [mg/cig] | 29.16 | 1.72 | BDL (0.067) | BDL | 99.89 | 30.94 | 1.13 | BDL (0.067) | BDL | 99.89 |
| Benzo(a)pyrene | [ng/cig] | 14.88 | 0.28 | NQ (0.354) | NQ | 98.28 | 14.15 | 0.15 | NQ (0.354) | NQ | 98.87 |
| 1,3-butadiene | [µg/cig] | 99.24 | 11.01 | NQ (0.095) | NQ | 99.94 | 92.91 | 10.16 | BDL (0.029) | BDL | 99.98 |
| Benzene | [µg/cig] | 83.16 | 0.49 | NQ (0.056) | NQ | 99.94 | 90.45 | 9.94 | NQ (0.056) | NQ | 99.95 |
| NNN | [ng/cig] | 322.67 | 19.88 | 27.43 | 2.47 | 91.50 | 308.40 | 19.67 | 20.36 | 2.57 | 93.40 |
| NNK | [ng/cig] | 303.78 | 14.33 | 6.62 | 0.87 | 97.82 | 256.00 | 12.81 | 4.77 | 0.52 | 98.14 |
| Formaldehyde | [µg/cig] | 44.13 | 5.34 | 1.91 | 0.12 | 95.68 | 39.89 | 1.61 | 1.30 | 0.18 | 96.75 |
| Acetaldehyde | [µg/cig] | 3129.40 | 178.12 | 175.19 | 8.99 | 94.40 | 1727.24 | 150.97 | 101.64 | 3.28 | 94.12 |
| Acrolein | [µg/cig] | 205.73 | 8.50 | 2.44 | 0.11 | 98.81 | 157.36 | 13.14 | 1.74 | 0.05 | 98.90 |
| **Aromatic Amines** |  |  |  |  |  |  |  |  |  |  |  |
| 1-aminonaphthalene | [ng/cig] | 20.88 | 2.01 | 0.03 | 0.01 | 99.85 | 22.93 | 0.73 | 0.04 | 0.00 | 99.84 |
| 2-aminonaphthalene | [ng/cig] | 16.30 | 0.88 | NQ (0.012) | NQ | 99.97 | 18.54 | 0.93 | NQ (0.012) | NQ | 99.96 |
| 3-aminobiphenyl | [ng/cig] | 5.19 | 0.45 | NQ (0.004) | NQ | 99.97 | 4.21 | 0.30 | NQ (0.004) | NQ | 99.93 |
| 4-aminobiphenyl | [ng/cig] | 3.40 | 0.25 | NQ (0.005) | NQ | 99.91 | 2.98 | 0.14 | NQ (0.005) | NQ | 99.91 |
| 2,5-dimethylaniline | [ng/cig] | 28.41 | 1.60 | NQ (0.020) | NQ | 99.93 | 25.82 | 0.89 | 0.02 | 0.01 | 99.92 |
| 2,6-dimethylaniline | [ng/cig] | 7.13 | 0.57 | 0.07 | 0.02 | 99.05 | 7.39 | 0.64 | 0.09 | 0.01 | 98.75 |
| Aniline | [ng/cig] | 545.28 | 26.10 | 3.82 | 1.32 | 99.30 | 524.70 | 21.16 | 1.86 | 0.16 | 99.64 |
| Benzidine | [ng/cig] | NQ (0.032) | NQ | BDL (0.003) | BDL | NC | BDL (0.010) | BDL | BDL (0.003) | BDL | NC |
| o-anisidine | [ng/cig] | 5.93 | 0.31 | 0.29 | 0.04 | 95.04 | 5.95 | 0.29 | 0.33 | 0.04 | 94.48 |
| o-toluidine | [ng/cig] | 106.99 | 4.91 | 0.32 | 0.03 | 99.70 | 112.56 | 6.32 | 0.43 | 0.03 | 99.61 |
| m-toluidine | [ng/cig] | 132.25 | 10.33 | 0.07 | 0.02 | 99.95 | 143.30 | 7.71 | 0.12 | 0.01 | 99.92 |
| p-toluidine | [ng/cig] | 88.69 | 8.99 | 0.07 | 0.02 | 99.92 | 93.03 | 5.79 | 0.11 | 0.01 | 99.88 |
