# Supplementary material for: Changes in Biomarkers of Exposure on Switching From a Conventional Cigarette to the glo Tobacco Heating Product: A Randomized, Controlled Ambulatory Study
Source: Nicotine Tob Res. 2020 Aug 10;23(3):584–91. doi: 10.1093/ntr/ntaa135 (PMC7885769; doi:10.1093/ntr/ntaa135)
Supplement: ntaa135_suppl_Supplementary_Table_2 [file ntaa135_suppl_supplementary_table_2.docx]

Supplementary Table 2. Biomarker of exposure (BoE) details for each BoE measured. LOD, limit of detection; LLOQ, lower limit of quantification; ULOQ, upper limit of quantification.

| **BoE** | **Abbreviation** | **Associated smoke constituent** | **Matrix** | **Units** | **LOD** | **LLOQ** | **ULOQ** |
| --- | --- | --- | --- | --- | --- | --- | --- |
| Total nicotine equivalents | TNeq | Nicotine | Urine | ng/ml | 0.5 | 1.5 | 5,000 |
| 4‑(methylnitrosamino)‑1‑(3‑pyridyl)‑1‑butanol | NNAL | NNK | Urine | pg/ml | 0.7 | 2.0 | 1,500 |
| N‑nitrosonornicotine | NNN | NNN | Urine | pg/ml | 0.2 | 0.5 | 1,500 |
| 3‑hydroxypropylmercapturic acid | 3-HPMA | Acrolein | Urine | ng/ml | 12.6 | 25 | 10,000 |
| 3‑hydroxy‑1‑methylpropylmercapturic acid | HMPMA | Crotonaldehyde | Urine | ng/ml | 1.5 | 5.0 | 2,500 |
| S‑phenylmercapturic acid | S-PMA | Benzene | Urine | ng/ml | 0.005 | 0.02 | 100 |
| Monohydroxybutenyl‑mercapturic acid | MHBMA | 1,3-butadiene | Urine | ng/ml | 0.043 | 0.129 | 10.34 |
| 2‑cyanoethylmercapturic acid | CEMA | Acrylonitrile | Urine | ng/ml | 0.08 | 0.25 | 1,250 |
| 4‑aminobiphenyl | 4‑ABP | 4-ABP | Urine | ng/l | 0.5 | 1.5 | 100 |
| *o*‑toluidine | *o*‑Tol | *o*‑Tol | Urine | ng/l | 0.8 | 10 | 1,000 |
| 2‑aminonaphthalene | 2‑AN | 2‑AN | Urine | ng/l | 0.6 | 1.7 | 100 |
| 1‑hydroxypyrene | 1‑OHP | Pyrene | Urine | ng/ml | 0.003 | 0.01 | 2 |
| 2‑hydroxyethylmercapturic acid | HEMA | Ethylene oxide | Urine | ng/ml | 0.05 | 0.2 | 100 |
| *N*-(2-cyanoethyl)valine (haemoglobin adduct) | CEVal | Acrylonitrile | Blood | pmol/g globin | 0.7 | 2 | 500 |
