# Supplementary material for: Changes in Biomarkers of Exposure on Switching From a Conventional Cigarette to the glo Tobacco Heating Product: A Randomized, Controlled Ambulatory Study
Source: Nicotine Tob Res. 2020 Aug 10;23(3):584–91. doi: 10.1093/ntr/ntaa135 (PMC7885769; doi:10.1093/ntr/ntaa135)
Supplement: ntaa135_suppl_Supplementary_Table_3 [file ntaa135_suppl_supplementary_table_3.docx]

Supplementary Table 3. Consumption Data for Study Participants. Data are the numbers of cigarettes smoked (Group A, continue to smoke) or numbers of Neostiks used (Group B, switch to glo) per day at timepoints up to day 90. Indicated days are ± 3 days due to individual subject visit scheduling. N, number of subjects; SD, standard deviation.

|  | Group | | | | | |
| --- | --- | --- | --- | --- | --- | --- |
|  | A (continue to smoke) | | | B (switch to glo) | | |
|  | Day 30 | Day 60 | Day 90 | Day 30 | Day 60 | Day 90 |
| N | 31 | 32 | 32 | 71 | 71 | 73 |
| Mean (SD) | 16.9 ± 5.1 | 17.5 ± 5.0 | 17.3 ± 5.4 | 19 ± 7.1 | 21 ± 8.3 | 21 ± 9.2 |
| Min | 2.5 | 10.6 | 10.0 | 9 | 8 | 4 |
| Max | 30.0 | 29.9 | 30.0 | 48 | 51 | 53 |
