# Supplementary material for: Changes in Biomarkers of Exposure on Switching From a Conventional Cigarette to the glo Tobacco Heating Product: A Randomized, Controlled Ambulatory Study
Source: Nicotine Tob Res. 2020 Aug 10;23(3):584–91. doi: 10.1093/ntr/ntaa135 (PMC7885769; doi:10.1093/ntr/ntaa135)
Supplement: ntaa135_suppl_Supplementary_Table_4 [file ntaa135_suppl_supplementary_table_4.docx]

**Supplementary Table 4. Between-Group Statistical Analysis of Change from Baseline (Day 1) to Day 90 in Biomarkers of Exposure in the Per Protocol Population.** Group A, continue to smoke combustible cigarettes; Group B, switch to glo. All analyses, except for eCO, were performed using biomarker levels from 24-h urine collections at baseline (day 1) and on day 90. Statistical analysis for the change in eCO levels were performed on data captured at baseline and the average of values obtained on days 120 and 150. TNeq, total nicotine equivalents (nicotine, cotinine, 3-hydroxycotinine and their glucuronide conjugates); HEMA, 2-hydroxyethylmercapturic acid; NNN, N-nitrosonornicotine; NNAL, 4-(methylnitrosamino)- 1-(3-pyridyl)-1-butanol; 3-HPMA, 3-hydroxypropylmercapturic acid; o-tol, o-toluidine; 4-ABP, 4‑aminobiphenyl; HMPMA, 3-hydroxy-1-methylpropylmercapturic acid; eCO, exhaled carbon monoxide; MHBMA, monohydroxybutenyl-mercapturic acid; 2-AN, 2-aminonaphthalene; S‑PMA, S-phenylmercapturic acid; CEMA, 2-cyanoethylmercapturic acid. LS mean, least squares mean; CI, confidence interval.

| **Biomarker (units)** | **Study Group** | **N** | **LS mean** | **Comparison** | **Difference (99.94% CI)** | **P value** |
| --- | --- | --- | --- | --- | --- | --- |
| **TNeq (mg/24h)** | Group A | 32 | -1.81 | B - A | -1.31 (-5.49, 2.87) | 0.27661 |
|  | Group B | 71 | -3.12 |  |  |  |
| **HEMA (µg/24h)** | Group A | 32 | -1.23 | B - A | -2.37 (-9.03, 4.3) | 0.21775 |
|  | Group B | 71 | -3.59 |  |  |  |
| **Total NNN (ng/24h)** | Group A | 32 | 2.15 | B - A | -8.96 (-24.43, 6.51) | 0.04493 |
|  | Group B | 71 | -6.81 |  |  |  |
| **Total NNAL (ng/24h)** | Group A | 32 | -12 | B - A | -108 (-168, -48) | <0.0001 |
|  | Group B | 71 | -120 |  |  |  |
| **3-HPMA (µg/24h)** | Group A | 32 | 218 | B - A | -925 (-1300, -551) | <0.0001 |
|  | Group B | 71 | -707 |  |  |  |
| **o-tol (ng/24h)** | Group A | 32 | -13 | B - A | -90 (-195, 14) | 0.00292 |
|  | Group B | 71 | -103 |  |  |  |
| **4-ABP (ng/24h)** | Group A | 32 | -1.3 | B - A | -11.5 (-16.3, -6.6) | <0.0001 |
|  | Group B | 71 | -12.8 |  |  |  |
| **HMPMA (µg/24h)** | Group A | 32 | -23 | B - A | -291 (-400, -182) | <0.0001 |
|  | Group B | 71 | -314 |  |  |  |
| **eCO (ppm)** | Group A | 32 | 14.98 | B - A | -12.47 (-17.25, -7.69) | <0.0001 |
|  | Group B | 72 | 2.51 |  |  |  |
| **MHBMA (µg/24h)** | Group A | 32 | 0.7 | B - A | -3.73 (-5.27, -2.18) | <0.0001 |
|  | Group B | 71 | -3.03 |  |  |  |
| **2-AN (ng/24h)** | Group A | 32 | -1.2 | B - A | -19.7 (-26.5, -12.9) | <0.0001 |
|  | Group B | 71 | -20.9 |  |  |  |
| **S-PMA (µg/24h)** | Group A | 32 | -0.49 | B - A | -3.14 (-4.52, -1.77) | <0.0001 |
|  | Group B | 71 | -3.64 |  |  |  |
| **CEMA (µg/24h)** | Group A | 32 | -4 | B - A | -147 (-191, -103) | <0.0001 |
|  | Group B | 71 | -151 |  |  |  |
