# Supplementary material for: Changes in Biomarkers of Exposure on Switching From a Conventional Cigarette to the glo Tobacco Heating Product: A Randomized, Controlled Ambulatory Study
Source: Nicotine Tob Res. 2020 Aug 10;23(3):584–91. doi: 10.1093/ntr/ntaa135 (PMC7885769; doi:10.1093/ntr/ntaa135)
Supplement: ntaa135_suppl_Supplementary_Figure_1 [file ntaa135_suppl_supplementary_figure_1.docx]

**Supplementary Figure 1. Mean Percentage Changes in Biomarkers of Exposure Between Baseline (Day 1) and Day 90 in the Per Protocol Population.** Data are mean values expressed as a percentage of the baseline value. All data, except for eCO, were calculated using biomarker levels from 24‑hour urine collections at baseline (day 1) and on day 90. eCO change was calculated from data captured at a single timepoint at baseline and on day 90. *n* = 32 (Group A), 71 (Group B) and 128 (Group D). TNeq, total nicotine equivalents (nicotine, cotinine, 3‑hydroxycotinine and their glucuronide conjugates); HEMA, 2‑hydroxyethylmercapturic acid; NNN, N‑nitrosonornicotine; NNAL, 4‑(methylnitrosamino)‑1‑(3‑pyridyl)‑1‑butanol; 3‑HPMA, 3‑hydroxypropylmercapturic acid; *o*‑tol, *o*‑toluidine; 4‑ABP, 4‑aminobiphenyl; HMPMA, 3-hydroxy-1-methylpropylmercapturic acid; eCO, exhaled carbon monoxide; MHBMA, monohydroxybutenyl‑mercapturic acid; 2‑AN, 2‑aminonaphthalene; S‑PMA, S‑phenylmercapturic acid; CEMA, 2‑cyanoethylmercapturic acid.
